# Essential oil-mediated biocompatible magnesium nanoparticles with enhanced antibacterial, antifungal, and photocatalytic efficacies

**DOI:** 10.1038/s41598-022-14984-3

**Published:** 2022-07-06

**Authors:** Diksha Pathania, Sunil Kumar, Pankaj Thakur, Vishal Chaudhary, Ajeet Kaushik, Rajender S. Varma, Hidemitsu Furukawa, Mamta Sharma, Ajit Khosla

**Affiliations:** 1 School of Biological and Environmental Sciences, Shoolini University, Solan, Himachal Pradesh 173212 India; 2 Department of Animal Sciences, Central University of Himachal Pradesh, Shahpur, Kangra, Himachal Pradesh 176206 India; 3 Special Center for Nanoscience, Jawaharlal Nehru University, New Delhi, 110067 India; 4 Research Cell and Department of Physics, Bhagini Nivedita College, University of Delhi, New Delhi, 110075 India; 5 NanoBio Tech Laboratory, Health System Engineering, Department of Environmental Engineering, Florida Polytechnic University, Lakeland, FL 33805-8531 USA; 6 School of Engineering, University of Petroleum and Energy Studies, Dehradun, Uttarakhand India; 7 Regional Centre of Advanced Technologies and Materials, Czech Advanced Technology and Research Institute, Palacky University in Olomouc, Šlechtitelů 27, 783 71 Olomouc, Czech Republic; 8 Department of Mechanical Systems Engineering, Graduate School of Science and Engineering, Yamagata University, Yonezawa, Yamagata 992-8510 Japan; 9 Department of Applied Chemistry, School of Advanced Materials and Nanotechnology, Xidian University, Xi’an, 710126 People’s Republic of China

**Keywords:** Green chemistry, Nanobiotechnology, Diseases

## Abstract

Emergent application of antimicrobial strategies as symptomatic treatment in coronavirus disease (COVID-19) and linkage of severe acute respiratory syndrome coronavirus2 with microbial infections, has created colossal demand for antimicrobials. For the first time, this communication explore the physicochemical, antifungal, antibacterial, and photocatalytic properties of biogenic magnesium nanoparticles (MgNPs), synthesized using essential oil of *Cymbopogon flexuosus's* as an efficient multifunctional reducing and stabilizing/capping reagent. It is observed that MgNPs (ranging in size: 8–16 nm) of varying phytochemical compositions (MgS1, MgS2, MgS3) exhibited various useful physicochemical, antimicrobial, and photocatalytic properties. FTIR outcomes highlight the functional biomolecules-assisted reduction of Mg from Mg^+^ to Mg^0^. Among all, MgS3-Nps owing to the smallest particle size exhibited superior photocatalytic efficacy (91.2%) for the methylene blue degradation upon direct exposure to the sunlight for 3 h without using any reducing agents. Fabricated MgNPs also exhibited excellent antifungal (against *Fusarium oxysporum*) and antibacterial (versus *Staphylococcus aureus* and *Escherichia coli*) efficacies compared to state-of-the-art antimicrobial agents deployed for the treatment of infectious diseases. Based on this investigated greener approach, imperative from economic and environmental viewpoint, such essential oil based-MgNPs can be a potential nanosystem for various industrial applications where photocatalytic, and biomedical attributes are the key requirements.

## Introduction

The severe outbreak of coronavirus disease 2019 (COVID-19) has created a global public health emergency and health management crisis^1^. The severity of this infection is increasing day by day as the severe acute respiratory syndrome coronavirus-2 (SARS-CoV-2) is mutating over time to generate more infectious and transmissible strains for example omicron, delta, etc.^2,3^. Therefore, successful COVID-19 management is still one of the biggest challenges to humanity till date^4,5^. The vicinity of COVID-19 cure treatment has resulted in emergent use of various antibiotics, antivirals, and antifungals to treat its secondary symptoms and control its severity. However, their efficacies, long term adverse effects, and safety have not been conclusively established and reported^6,7^. Severe acute respiratory syndrome coronavirus 2 (SARS-CoV-2) is responsible for COVID-19 pandemic and linked to numerous bacterial and fungal infections. For instance, patients with erroneous administration of several corticosteroids (i.e., Prednisone, Hydrocortisone, or Dexamethasone) to cure inflammation are infected with lethal pathogenic infection like mucormycosis^8^. The inclusion of antimicrobial drugs and spread of secondary infections has raised the requirement of biocompatible antifungal, antiviral and antibiotic based on nanomaterials^9^. Moreover, the photocatalytic efficacies of these nanomaterials can be further utilized to design smart strategies like photocatalytic paint with self-cleaning ability to combat infections. For instance, Hassanpour et al*.* synthesized Co_3_O_4_/ZnO heterojunction through green method for the degradation of RhB and MB dye within 90 min under UV–visible light irradiation^10^. Co_3_O_4_/ZnO heterojunction photodegraded 90 and 80% of RhB and MB dye within 90 min.

Interestingly, metal based nanomaterials possess excellent antimicrobial and photocatalytic efficacies owing to their unique physicochemical properties and bio-reductant activities^11,12^. Traditionally, silver, zinc, nickel^13^, titanium^14^ and copper-based nanoparticles are utilized for antimicrobial and photocatalytic applications^15,16^. However, their biocompatibility, toxicity, and role as source of nano waste are bottlenecks, which limits their commercial prospects in nanomedicines, agriculture, and food industries.

Contrary, magnesium (Mg)-based NPs are biocompatible, considered comparatively safer for animals^17^, and is a crucial element for plant development and photosynthesis. The Food and Drug Administration (FDA) from the U.S. has recognized Mg-based NPs as a safe alternative with exceptionally efficient antimicrobial activities^18,19^. It is attributed to unique and stable physicochemical characteristics of Mg NPs including high optical and electrical band gap, thermodynamic stability, low dielectric constant, and low refractive index. Mg-based NPs are also used to diagnose and treat several diseases, such as heartburn diseases where it acts as an anti-acid and stomach pain, among others^20^. These unique features of MgNPs have culminated in enormous scientific work to manufacture, characterize, and utilize nanoparticles^21^.

Primarily, Mg NPs are commercially fabricated utilizing top-down (physical) and bottom-up (chemical) strategies like ultrasonication, microwave irradiation, wet impregnation, laser-vaporization routes, sol–gel, and hydrothermal routes. However, the inclusion of hazardous chemicals deployed in the synthesis of Mg-based NPs increases their toxicity and contaminates the environment^22^. On the other hand, physical approaches require a large amount of energy, turning them into less economical techniques.

In contrast, biological strategies termed as 'greener synthesis’ alternatives as they tend to be cost-effective, environmentally friendly, less toxic, and often biocompatible^10^. These routes includes bio-reductants extracted using plants, bacteria, fungi, yeasts, micro-and macro-algae, and other natural resources^23^.

Plant extracted essential oils are preferred over other natural resources due to the abundance of biomolecules and phytochemicals, convenience of extraction, easy availability and management, relatively low cost, high bio-reducing efficacy, biosafety and rapidity of reaction rates that make them attractive proposition^24^. Numerous Phyto molecules present in natural plant extracts serve as effective reducing, capping, and stabilizing agents during the assembly of NPs, which eliminates the separate requirement of stabilizers, reductants, and surfactants. These bioactive metabolites like terpenoids act as natural bio-reductant for metal ions and concurrently stabilizes the NPs as capping agents by reducing the direct contact between molecules^25,26^.

*Cymbopogon flexuosus*, commonly known as lemongrass, is a popular choice for deployment in green synthesis and is used chiefly for cough, cold, and fever as a traditional medicinal remedy. Fascinatingly, it helps to increase the immune system, decreases uric acid, cholesterol, extra fat, and alleviates gastroenteritis and indigestion. Recent scientific research has examined lemongrass's essential oil for its impact on cancer cells as it decreases the viability of the tumor cells via apoptotic activation attributable to its main component ‘Citral’, which has strong anticancer characteristics^27^. However, numerous bottlenecks like poor physical properties such as hydrophobicity, susceptible to degradation and volatility restricts their commercial use in pharmaceutical applications. These limitations have been addressed to an extent by encapsulating these essential oils into a nanocarrier, helpful in-target delivery and controlled release at the disease site^28^. The utilization of lemon grass essential oil to fabricate Mg NPs is anticipated to include merits of the precursors in a single nanomaterial^29^.

With this motivation, this unprecedented study investigates the physicochemical, antibacterial, antifungal, and photocatalytic properties of *C. flexuosus* essential oil mediated Mg NPs. The physicochemical characteristics of fabricated biogenic Mg NPs due to different phytochemical compositions of essential oil are explored through various spectroscopic investigations. The antimicrobial activities of fabricated Mg NPs were evaluated against two bacterial strains (one positive *S. aureus* and one negative *E. coli*) and a fungal strain (*F. oxysporum*) while their photocatalytic degradation efficiency was assessed for methylene blue (MB) dye under direct sunlight.

## Result and discussion

### Structural evaluation: UV–visible absorption spectroscopy

**Figure 1:**
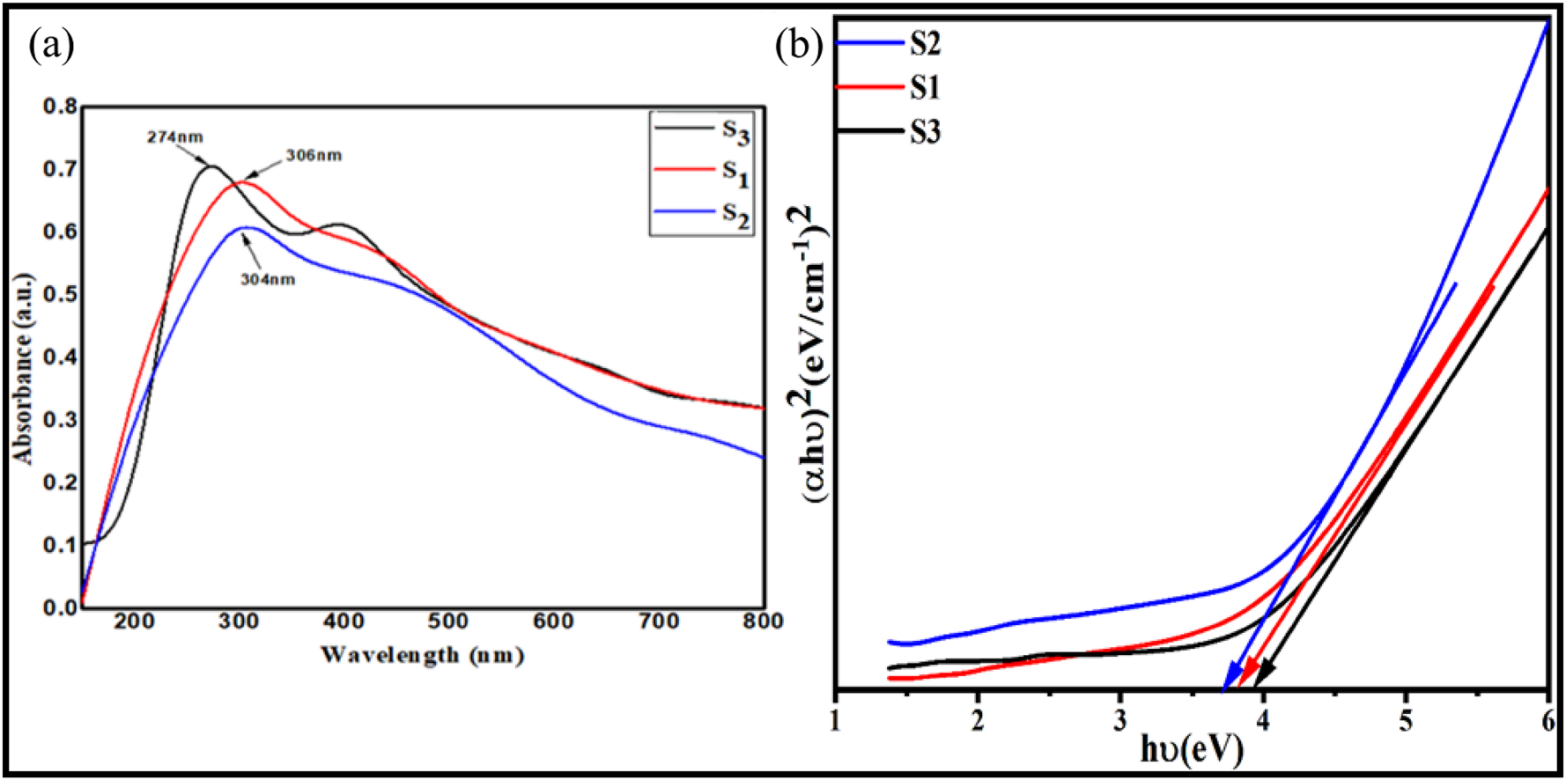
(**a**) UV–visible spectra and (**b**) Tauc plot of Mg NPs using essential oil extracts of *C*. *flexuosus* from different altitudes, i.e., MgS1, MgS2, and MgS3.

The initial verification of the formation of the MgNPs was acquired by the decrease in the color intensity of the essential oil. The alteration in color from light yellow to pale yellow suspension verified the bioreduction process converting magnesium ions into MgNPs. Apart from the color conversion, the appearance of a specific absorption band affiliated with the MgS1, MgS2, and MgS3 at 306 nm, 304 nm, and 274 nm affirmed the formation of the MgNPs via the deployed greener route (Fig. 1a). The appearance of a single peak in the MgS1, MgS2, and MgS3 Nps spectrum proposes that the fabricated NPs have small-sized nanoparticles^30^, which is further validated using spectroscopic techniques. The presence of band-edged at the lower wavenumber side proposes faster photocatalytic interaction of S3 than that of other samples. Using Tauc plot, band gap of MgS1, MgS2, and MgS3 is around 3.82, 3.76 and 3.96 eV respectively (Fig. 1a, b).

### FTIR analysis to evaluate functional properties of MgNPs

**Figure 2:**
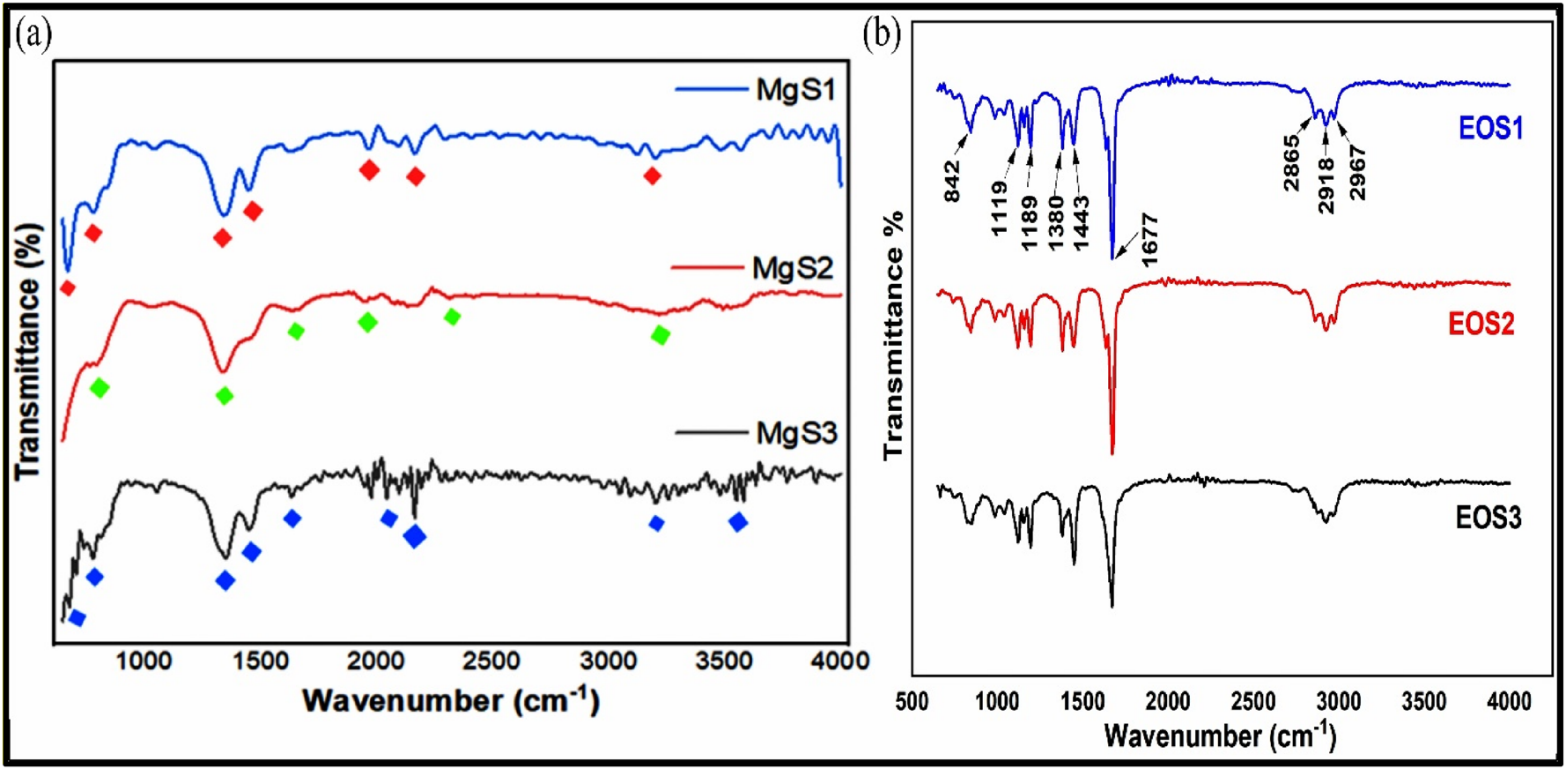
FTIR spectra for (**a**) MgNPs and (**b**) essential oil of *C*. *flexuosus* from different altitudes.

FTIR analysis was utilized to depict the functional role of phytochemicals present inessential oil as capping agents and in stabilizing the fabricated NPs as shown in Fig. 2a. FT-IR for Mg S1, Mg S2, and Mg S3 confirmed the presence of prominent characteristic peaks (Table 1) for all the fabricated samples at 3563 cm^−1^ 3486 cm^−1^, 3210 cm^−1^, 3203 cm^−1^ and 3150 cm^−1^. They correspond to the presence of O–H group due to hydrogen bonds arising from NH_2_ and OH groups in protein molecules, and the presence of citral and related terpenoids content. The peaks at 1957 cm^−1^, 1961 cm^−1^, 987 cm^−1^ in samples Mg S1, Mg S2 and Mg S3, respectively represents the aromatic combination bands. The absorbance peaks at 1629 cm^−1^ and 1457 cm^−1^ in S1, at 1640 cm^−1^ and 1448 cm^−1^ in S2, at 1633 cm^−1^ and 1457 cm^−1^ in S3, indicates the bending mode of N–H bond with either amide or carboxylate salt. The bending of C–H band vibrations of aromatic amines is observed at ~ 1343 cm^−1^ in Mg S1 and Mg S2, and at 1358 cm^−1^ in Mg S3. In Mg S1, Mg S2 and Mg S3, transmission peaks at 786 cm^−1^ and 685 cm^−1^ correspond to the formation of nanoscale Mg NPs. The slight shift of absorbance peaks towards lower wave number is attributed to the presence of constituents in the essential oil of *C. flexuosus*. Furthermore Alkene, primary amide, amine, aldehyde, ketone and ethers were found in the produced samples by FTIR. According to the literature and the FTIR spectra of essential oil (Fig. 2b), these functional groups have been found. Some of these compounds included alkenes, alcohols (cis Verbenol, geraniol), ketones (4-Nonanone), and aldehydes (Citral)^31^. The shifting of characteristic peaks is due to constituents of essential oil which functioned as reducing and stabilizing agents. Finally, the FTIR spectra revealed the presence of all the characteristic peaks which endorses the structure of NPs and illustrates the role of functional groups of phytochemicals as reducing, capping and stabilizing reagents for architecting NP structure.

**Table 1:** Position of characteristic stretching modes in FTIR spectra of MgS1, MgS2, MgS3 NPs.

Stretching bonds →	Metal–oxygen bond (cm^−1^)	C–O stretching bond (cm^−1^)	C–H band vibrations (cm^−1^)	N–H bond (cm^−1^)	Aromatic combination bands (cm^−1^)	Hydrogen bonds O–H group (cm^−1^)
Mg S1	786 and 685	1043	1343	1629 and 1457	1957	3480 and 3150
Mg S2	786 and 685	1043	1343	1640 and 1448	1961	3486 and 3210
Mg S3	786 and 685	1047	1358	1633 and 1457	1987	3563 and 3203

### Particle size and structural analysis of MgNPs

**Figure 3:**
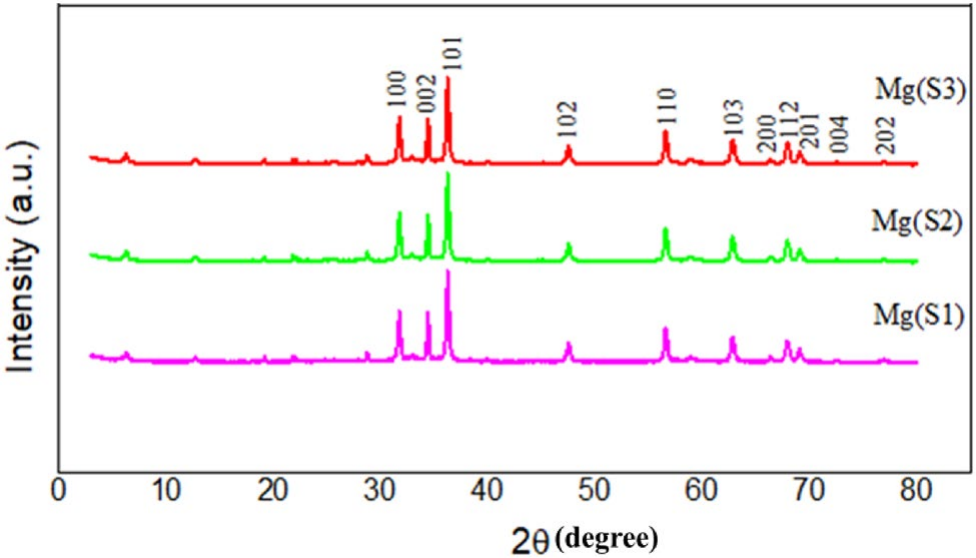
X-ray diffraction for MgNPs using essential oil of *C*. *flexuosus* from different altitudes.

The purity, size, and crystalline structure of the biofabricated MgNPs have been determined by XRD (X-ray diffraction technique) as shown in Fig. 3.The eleven divergent diffraction peaks at 31.74°, 34.45°, 36.32°, 47.48°, 56.52°, 62.75°, 66.38°, 67.92°, 69.09°, 72.50° and 76.96° were corresponding to the (100), (002), (101), (102), (110), (103), (200), (112), (201), (004), and (202), respectively. All the diffraction peaks could bereadily indexed to various crystal planes of the hexagonal phase of Mg nanoparticles (JCPDS file no. 04-0770). No peaks from another phaseswere detected, indicating that the product synthesized by the route is of high purity. The average crystalline size of nanoparticles corresponding to the most intense diffraction peak at 2θ = 36.32° (101) as calculated by the Debye–Scherrer equation for MgS1, MgS2, and MgS3 was found to be 13.21 nm, 12.90 nm, and 11.35 nm, respectively. XRD results confirmed that MgNPs are highly crystalline, having a hexagonal wurtzite structure. Small humps are also observed due to the presence of organic molecules (generally amorphous) due to the presenceof essential oil during the fabrication.

**Figure 4:**
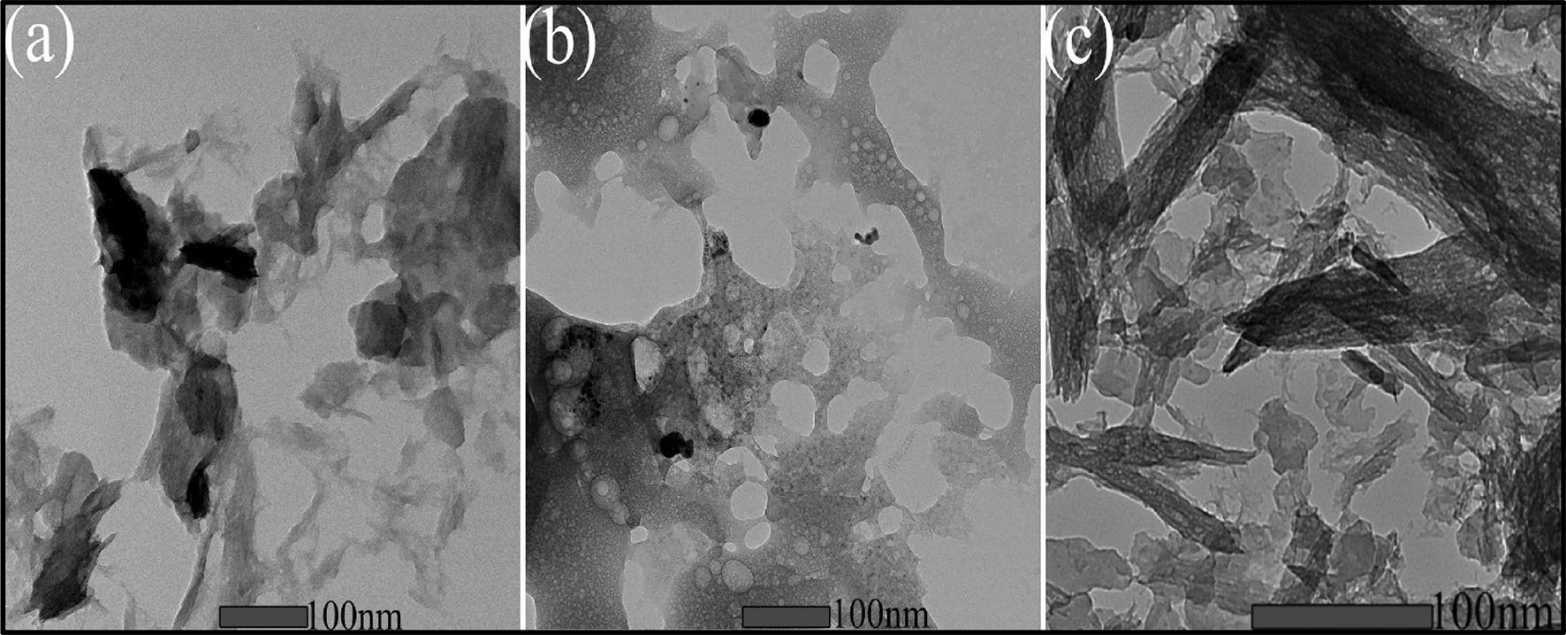
TEM analysis for MgNPs using essential oil of *C*. *flexuosus* from different altitudes of (**a**) MgS1, (**b**) MgS2, and (**c**) MgS3.

The particle size was further established by using transmission electron microscopy (TEM) and images are displayed in Fig. 4a–c, which revealed the formation of aggregated Mg NPs. The range of biofabricated MgNPs was in the range of 8–90 nm in diameter, which is consistent with XRD results. Such low dimensional nanoparticles possess high surface to volume ratio anticipating high surface reaction-based usages like antimicrobial and photocatalytic activities.

**Figure 5:**
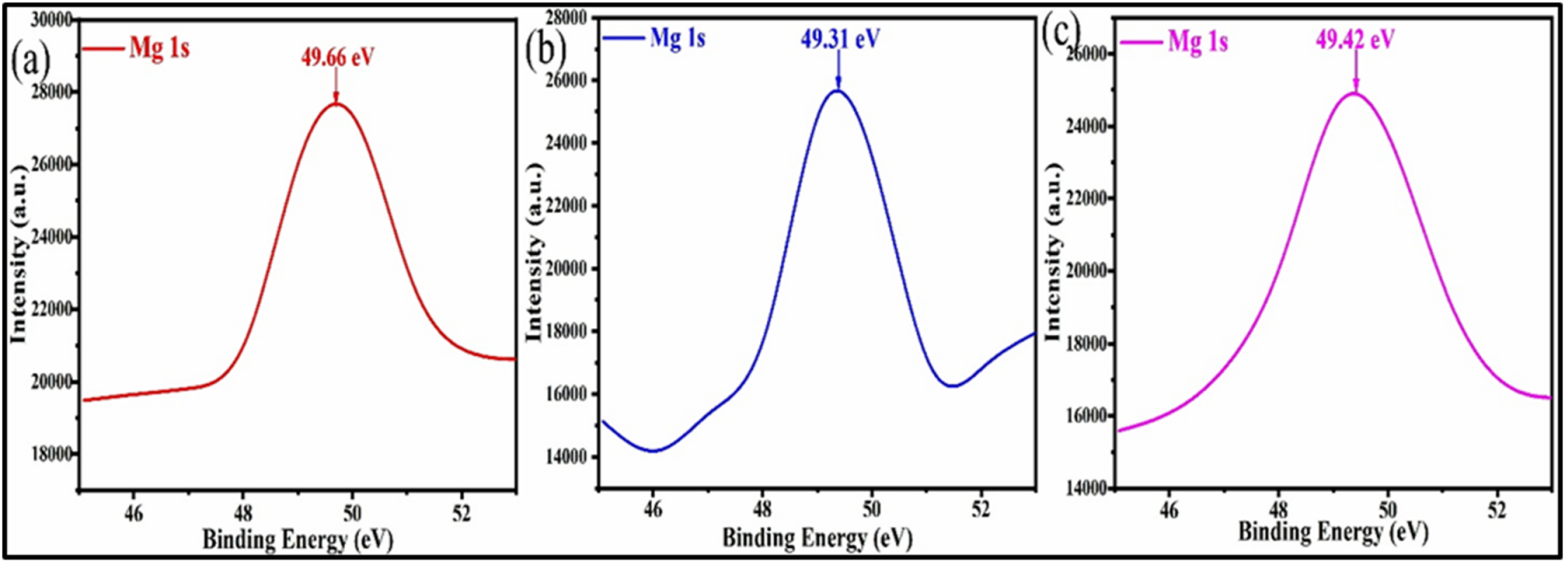
XPS spectra of (**a**) MgS1, (**b**) MgS2, (**c**) MgS3.

XPS analysis of Mg S1, MgS2, MgS3 showed Mg 1 s spectra (Fig. 5a–c). MgS1, MgS2, and Mg S3 showed peak at 49.66 eV, 49.31 eV, and 49.42 eV respectively. This is in excellent accord with information originally collected for Mg(OH)_2_^32^. Due to moisture in air, Mg(OH)_2_ is likely to be the most common species on the surface of Mg NPs.

### Evaluation of photocatalytic activity of MgNPs

**Figure 6:**
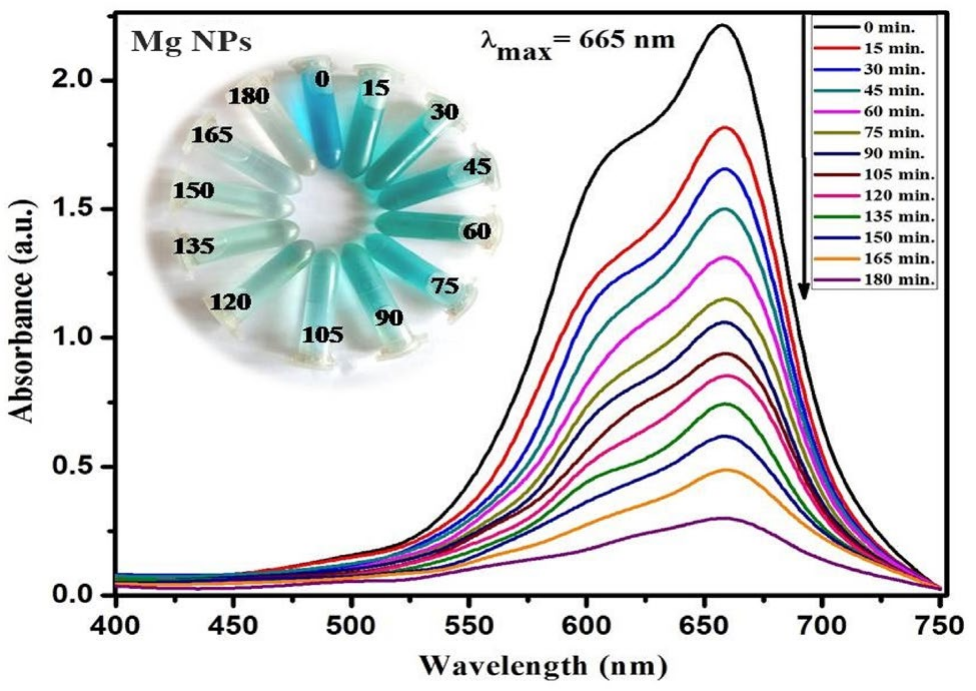
UV–vis absorption spectra of MB dye using Mg NPs.

**Figure 7:**
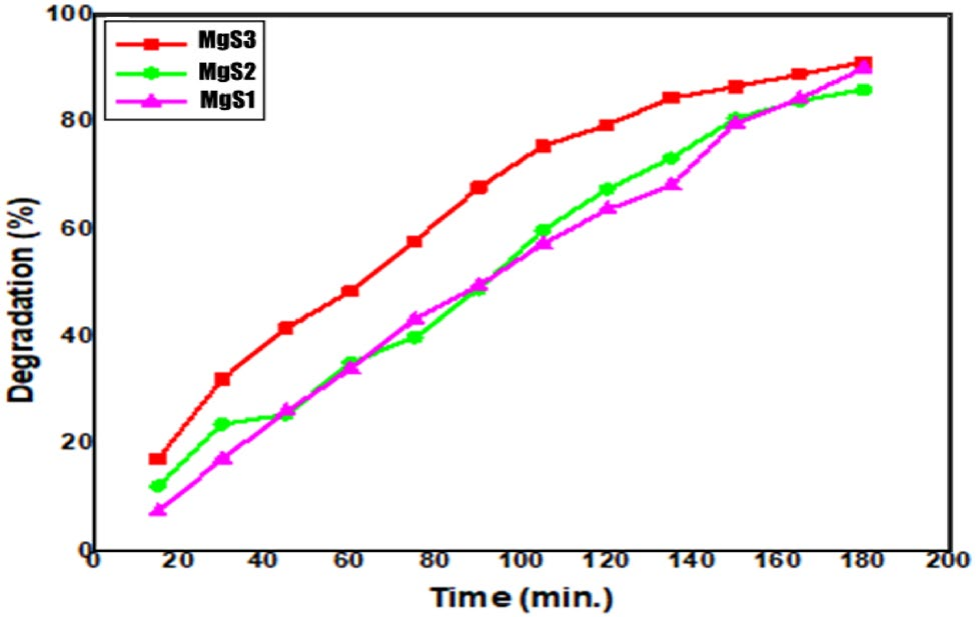
Photodegradation percentage of MB using Mg NPs fabricated from the essential oil of *C. flexuosus* for MgS1, MgS2 and MgS3.

**Figure 8:**
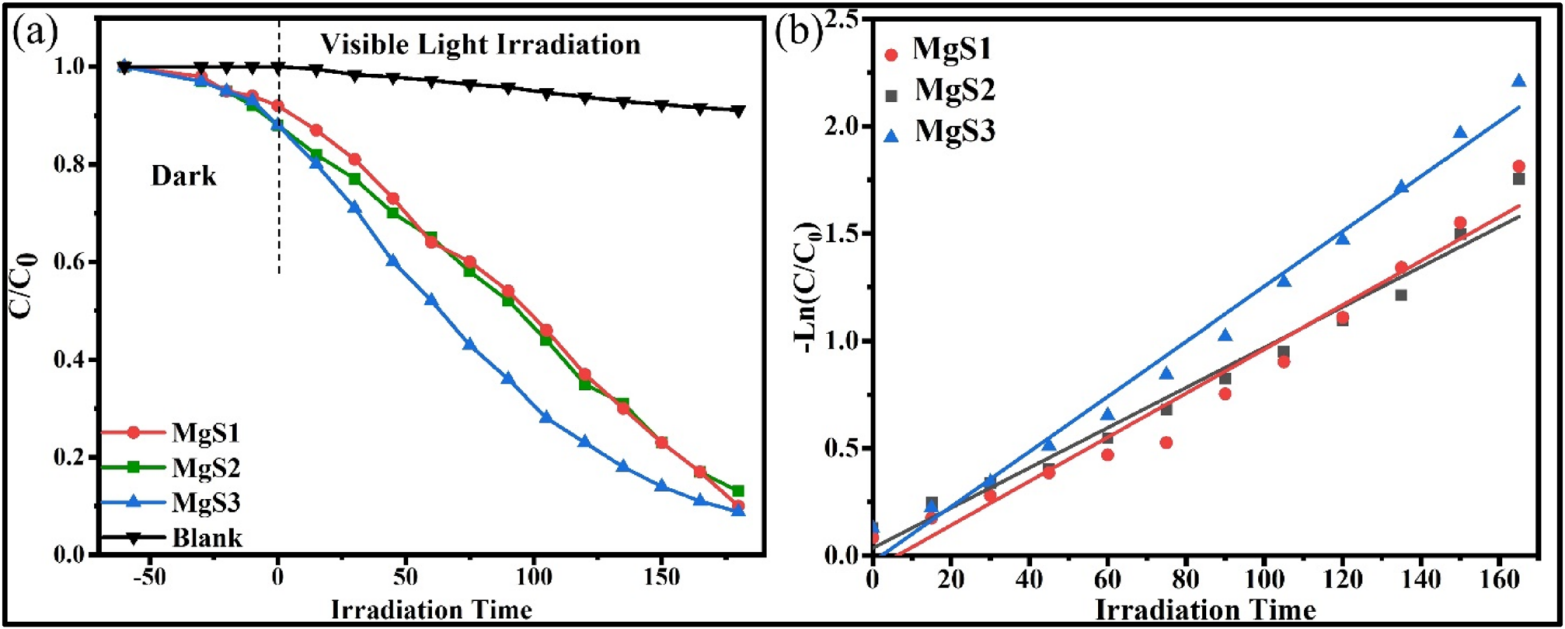
(**a**) C/C_0_ versus time graph and (**b**) pseudo-first order kinetics fitting data for photo-degradation for MB degradation using Mg NPs fabricated.

MgNPs were investigated for their catalytic efficacy in the photodegradation of organic methyl thioninium chloride, commonly known as Methylene Blue (MB). Its redox properties have several medical applications, including the treatment of methemoglobinemia, smooth muscle relaxation, near-infrared fluorescence (NIR) fluorescent dye, and surgical procedures. Despite being widely using MB as a human therapeutic agent, its detrimental environmental impact has raised serious concerns; degradation of MB dye has been carried out using sunlight as a natural activator^33^. Fabricated Mg NPs utilizing *C. flexuosus* essential oil have shown remarkable photodegradation activity against methylene blue polluted water samples at various degradation intervals. The decrease in peak absorbance of MB at 665 nm as a function of irradiation time depicts the photocatalytic efficacy of applied Mg NPs (Fig. 6). It depicts the gradual decline in absorption peak intensity within initial 15 min, which eventually disappeared entirely at the end of 180 min. Figure 7 presents the time profile of sunlight-assisted MB dye degradation efficiency. Within 60 min of photocatalysis, ~ 48.4% in Mg S3 (which is highest among all the sites), 35.1% in Mg S2, and 34% in Mg S1 of the total MB concentration were degraded, yielding a final MB degradation efficiency of 91.2% for MgS3 (maximum), 90.1% for MgS1, and 86.1% forMgS2 within 180 min. MgS3 Nps showed the highest degradation efficiency due to the low band edge and smallest particle size, which illustrates the generation of radicals during photocatalysis. Figure 8a revealed the C/C_0_ versus time reaction for MB degradation under visible light irradiation. Pseudo-first-order kinetics was achieved by graphing linear ln (C/C_0_) versus time, as shown in (Fig. 8b). The high concentration gradient and numerous catalytic sites encouraged the fast color deterioration during the initial phase of degradation. The photocatalytic degradation of MB confirms the amphoteric nature of Mg NPs. In the presence of sunlight, the photodegradation capability of Mg NPs anticipated a three-step mechanism that involves the generation and transfer of electron–hole pairs, radical generation, and then degradation of dye. The electrons and holes generated from magnesium conduction and valance bands, respectively in the first step by irradiating the material with sunlight, as explained in Eq. (1).

1 $${\text{Mg}} + {\text{h}}\upnu \to {\text{e}}^{ - }_{{{\text{cb}}}} + {\text{h}}^{ + }_{{{\text{vb}}}}$$

The recombination rate of charge carriers is decreased with sunlight irradiation and facilitating electron–hole pair separation on the surface. In the second phase, the oxygen molecule interacts with surface electrons to generate superoxides, converted to peroxide molecules. In addition, the surface holes oxidize the water molecules, resulting in the formation of hydroxyl ions, as shown in Eqs. (2)–(4).

2 $${\text{O}}_{2} + {\text{e}}^{ - } \to {\text{O}}^{^\circ }_{2} + {\text{H}}^{ + } \to {\text{HOO}}^{^\circ }$$

3 $${\text{HOO}}^{^\circ } + {\text{e}}^{ - } + {\text{H}}^{ + } \to {\text{H}}_{2} {\text{O}}_{2}$$

4 $${\text{H}}_{2} {\text{O}} + {\text{h}}^{ + } \to {\text{OH}}^{^\circ } + {\text{H}}^{ + }$$

The above-formed intermediates are exceedingly unstable, and when they react with the dye substituent, it disintegrates into mineralized products, as presented in Eq. (5).

5 $${\text{MB}}\;{\text{Dye}}\;{\text{molecule}} + \left( {{\text{OH}}^{^\circ } ,{\text{HOO}}^{^\circ } ,{\text{H}}^{ + } \;{\text{or}}\;{\text{H}}_{2} {\text{O}}_{2} } \right) \to {\text{Intermediate}}\;{\text{products}} \to {\text{CO}}_{2} + {\text{H}}_{2} {\text{O}}$$

However, the dye degradation efficiency of Mg NPs is dramatically lowered due to severe aggregation. The findings of photodegradation investigations revealed that as-fabricated Mg NPs have significant photocatalytic activity. The almost total disintegration of MB, with ~ 91.2% photodegradation within 3 h, indicates that prepared Mg NPs had substantial photocatalytic efficiency compared to that of reported in literature. For instance, Ravichandran et al. fabricated Ag nanoparticles using *Parkia speciosa* leaf aqueous extract and observed 84% degradation of methylene blue within 180 min under sunlight irradiation^34^.

Further analysis revealed that the photocatalytic efficacy of Mg S3Nps is superior to that of other materials, which is attributed to its smaller particle size (as observed from TEM & XRD results) and lower band edge (as obtained from UV results). The smaller size particles possess a larger surface-to-volume ratio, which enhances the surface interaction between the direct sunlight and the particle's surface, resulting in high photocatalytic efficacy. The results are further summarized in Table 2.

**Table 2:** Sunlight assisted photocatalytic performance of fabricated Mg NPs in terms of physicochemical characteristics.

Samples	Band edge (eV)	Crystallite size (TEM) (nm)	Crystallite size (XRD) (nm)	Photo-catlytic efficiency (%)
MgS1	2.68	12–80	13.21	90.1
MgS2	2.72	10–90	12.90	86.1
MgS3	2.55	8–70	11.35	91.2

### Assessment of antibacterial efficiency of MgNPs

**Figure 9:**
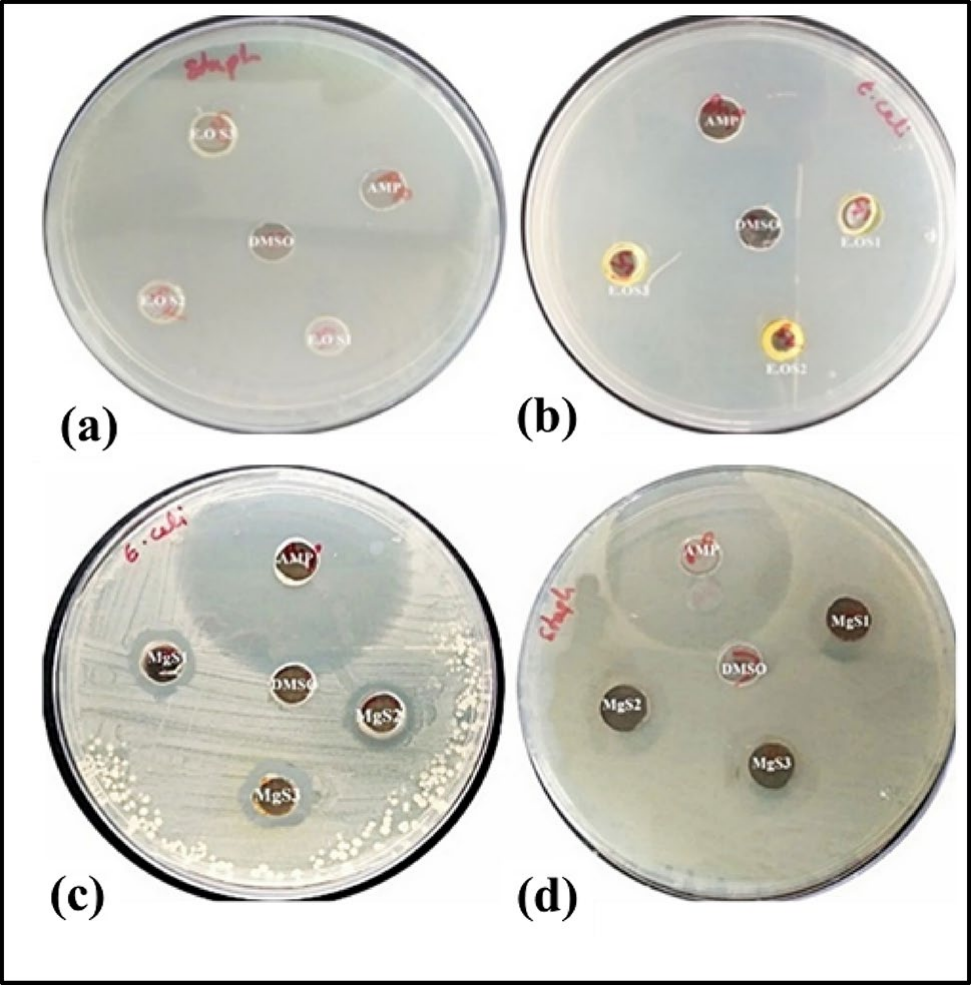
Antimicrobial activity of Essential oil against (**a**) *S. aureus* and (**b**) *E. coli* from different altitudes (S1-Palampur, S2-Haryana, and S3-Dehradun). Antimicrobial activity of Mg NPs against (**c**) *E. coli* and (**d**) *S. aureus* from different altitudes (MgS1-Palampur, MgS2-Haryana, MgS3-Dehradun).

The antimicrobial potential of MgNPs and extracted essential oil was assessed against various multi-drug-resistant microorganisms. Antibacterial activity of biofabricated MgNPs from the essential oil of *C. flexuosus* has been studied against two pathogenic bacteria i.e., gram + ve (*S. aureus*) and gram − ve (*E. coli*), using well diffusion assay and zone of inhibition as depicted in Fig. 9a–d. Wells were loaded with the same concentration (80µL) of synthesized Mg NPs from different altitudes.

**Figure 10:**
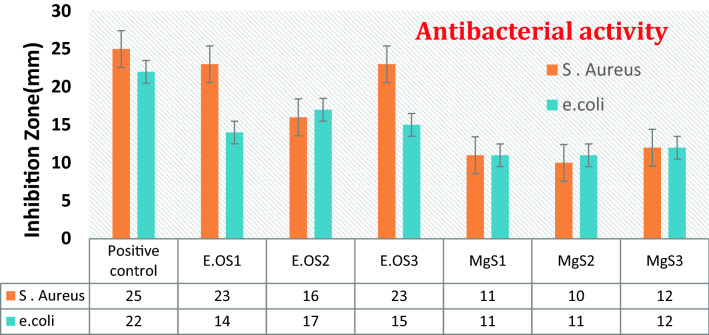
Antibacterial activity of *C. flexuosus* (E.OS1, E.OS2 and E.OS3) essential oil and synthesized MgNPs (MgS1, MgS2, and MgS3) against *S. aureus and E. coli*.

For Mg NPs from essential oil extract S1 (Palampur) and S3 (Dehradun), maximum zone of inhibition (23 mm) has been observed against *S. aureus* and the minimum was observed for S2 (Haryana) (16 mm). Against *E. coli, a* maximum zone of inhibition (17 mm) is observed from the essential oil of S2 (Haryana), and a minimum zone of inhibition (15 mm) has been observed for S3 (Dehradun). However, MgNPs synthesized from essential oil extract S3 showed maximum zone of inhibition (12 mm) against *S. aureus*, and the minimum zone of inhibition(10 mm) was observed forS2 (Haryana) (Fig. 10). On the other hand, against *E. coli,* maximum zone of inhibition (12 mm) was seen from MgNPs synthesized from the essential oil of S3 (Dehradun) and a minimum zone of inhibition (11 mm) has been observed for MgS2 (Haryana) along with S1 (Palampur) respectively. The enhanced antibacterial efficacy of the prepared samples is attributed to their smaller particle size.

Table 3 summarizes the antimicrobial potential of fabricated Mg NPs and essential oil only against some prominent pathogenic indicator strains. The obtained results reveal that the essential oil has excellent antibacterial activity against gram-positive and gram-negative bacteria. Fabricated MgNPs also exhibited notable antibacterial effects as a biocidal agent, which is ascribed to their smaller particle size and increased surface area. It is anticipated that the microbial cells might have quickly taken up the distinct nanoscale-sized MgNPs. Similarly, nanoparticles displayed a much profound effect on all selected microorganisms. It is attributed to presence citral in lemongrass essential oil, which is as an active compound known for its biocidal activities. Fatiqin et al*.* fabricated MgO nanoparticle through green synthesis using *Moringa oleifera* leaf extract for the antibacterial activity against *S. aureus and E. coli*^35^. MgO nanoparticles revealed 6.3 mm and 6 mm zone of inhibition for *S. aureus and E. coli* respectively. Therefore, essential oil-mediated nanoparticles showed significant antimicrobial efficacies and were well-suited for drug applications^36^.

**Table 3:** Antibacterial activity of *C. flexuosus* (E.OS1, E.OS2 and E.OS3) essential oil and synthesized MgNPs (MgS1, MgS2, and MgS3) against *S. aureus and E. coli*.

Samples	Microorganisms	
	*S. aureus* (mm)	*E. coli* (mm)
Positive control	25	22
E.OS1	23	14
E.OS2	16	17
E.OS3	23	15
MgS1	11	11
MgS2	10	11
MgS3	12	12

### Assessment of antifungal efficiency of MgNPs

**Figure 11:**
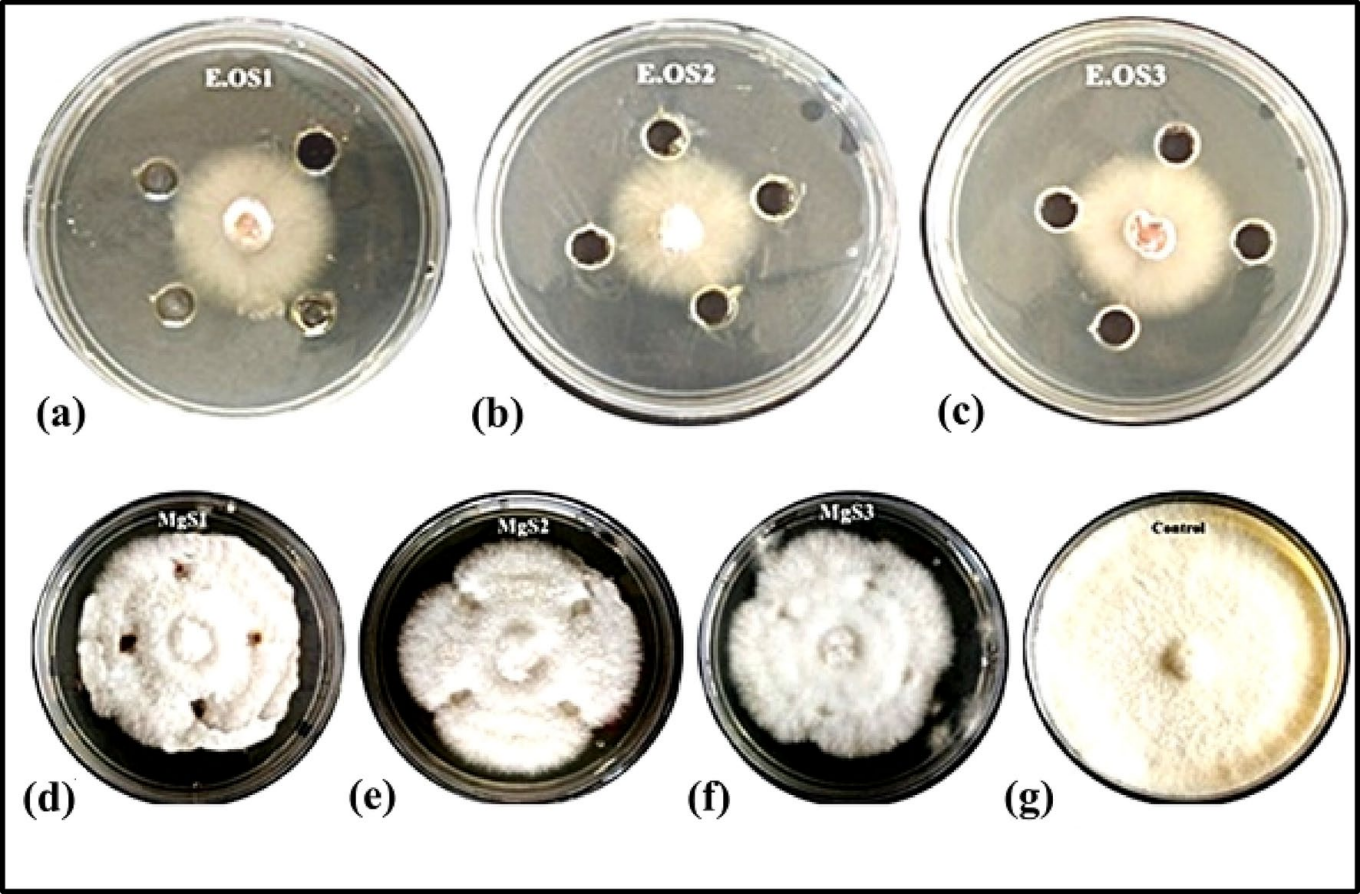
Antifungal activity of *C. flexuosus* essential oil against: *F. oxysporum* from different altitudes (**a**) E.OS1, (**b**) E.OS2, (**c**) E.OS3. Antifungal activity of Mg NPs against: *F. oxysporum* from different altitudes (**d**) MgS1, (**e**) MgS2, (**f**) MgS3, and (**g**) control.

The extracted essential oil of *C. flexuosus* and fabricated MgNPs were also evaluated for antifungal performance against *F. oxysporum* using the good diffusion method and zone of inhibition as depicted in Fig. 11a–g; wells were loaded with the same concentration (60 µL) of fabricated Mg NPs.

**Figure 12:**
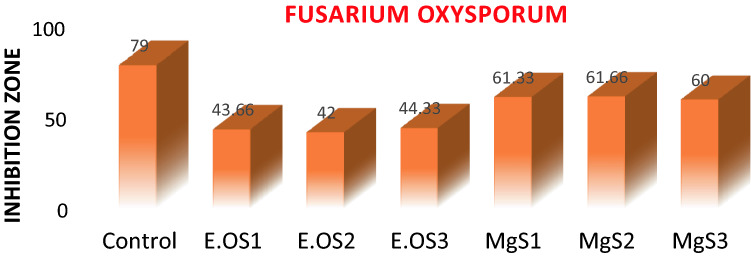
Antifungal activity of *C. flexuosus* (E.OS1, E.OS2, and E.OS3) essential oil and synthesized MgNPs (MgS1, MgS2, and MgS3) against *F. oxysporum*.

**Figure 13:**
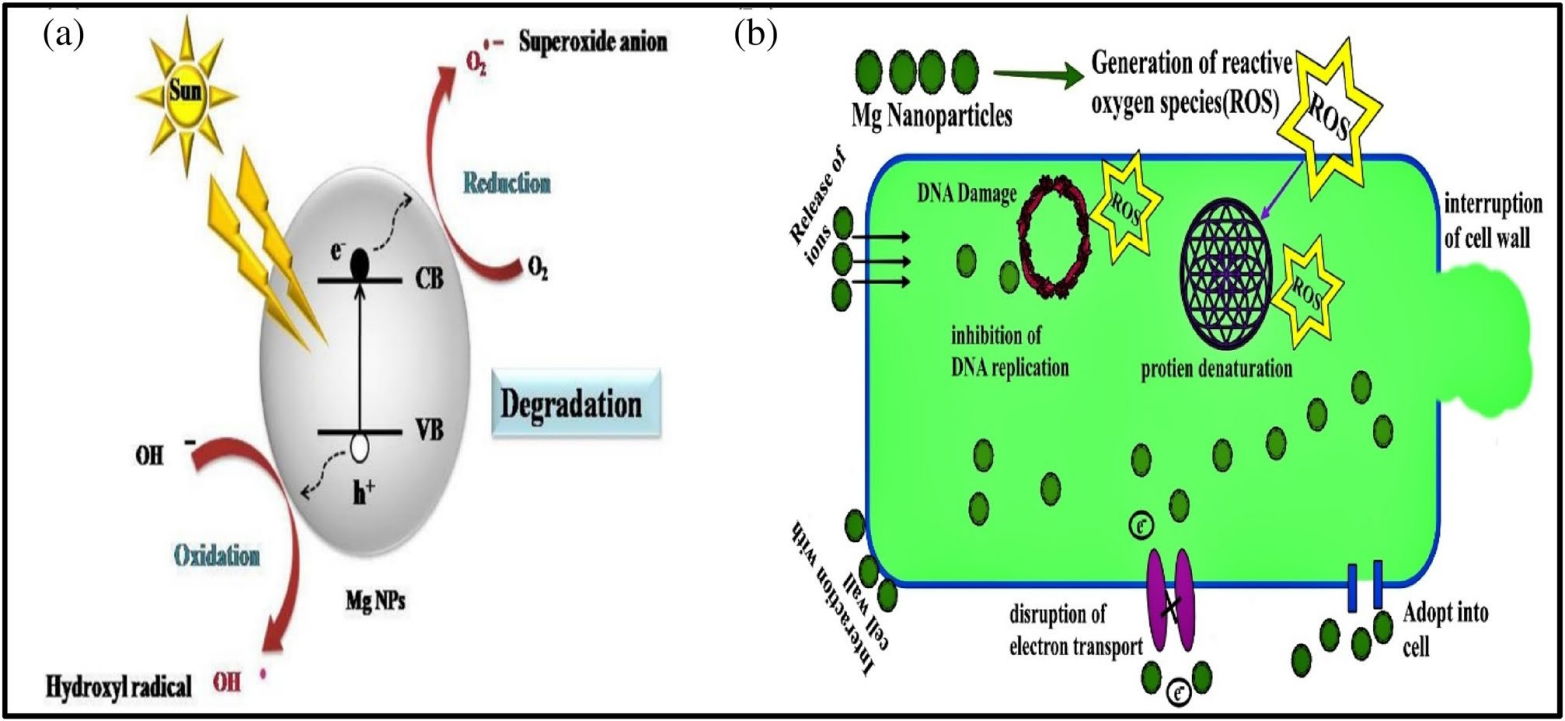
Schematic mechanism of (**a**) photocatalytic degradation, (**b**) antimicrobial activity by Mg nanoparticles.

The magnitude of the antifungal agent was determined based on the size of the inhibition zone formed around each well loaded with an appropriate test sample. Table 4 and Fig. 12 shows the diameter of the inhibition zone formed around wells and the percentage inhibition. Even though all samples exhibited antifungal activity, the MgS2 showed the highest activity, 46.83% of inhibition. The antifungal activity of MgNPs generally depends on the presence of higher ROS that generally comes from a better photocatalyst with larger surface area, crystallite size, increased oxygen vacancies, diffusion ability of the reactant molecules, and the release of Mg^2+^. As mentioned earlier, the more significant ROS role is mainly attributed to the larger surface area, increase in oxygen vacancies, and the reactant molecules' diffusion ability. Magnesium ions (Mg^2+^) have antimicrobial effect against a variety of fungal and bacterial strains because Mg nanoparticles release Mg^2+^ ions into aqueous solution, as illustrated in Fig. 12. In the present investigation, the antifungal effect of the MgS2 samples is mainly due to the combination of various factors such as essential oil constituents, ROS, and the release of Mg^2+^; photocatalysis appears to be the most crucial antifungal mechanism (Fig. 13). ROS produced on the surface of these NPs in the presence of light causes oxidative stress in microbial cells and eventually leads to the cell's death. ROS contains the most reactive hydroxyl radical (^·^OH), less toxic superoxide anion radical (^·^O_2_^−^), and hydrogen peroxide with a weaker oxidizer (H_2_O_2_), which can penetrate the cell membrane and kill the microbes^37^. Sharmila et al*.* synthesized MgO nanoparticles through green synthesis using leaf extract of *Pisonia alba* for antifungal activity against *Aspergillus flavus* and *Fusarium solani*^38^. MgO nanoparticles showed maximum 4 mm and 3 mm zone of inhibition against *Aspergillus flavus* and *Fusarium solani*. Moreover, the smaller particle size, especially in the nanoscale further enhances their antifungal efficacies due to increases surface interaction amongst fungus and particles, and optimum photocatalytic activity. The antifungal activity of essential oil and MgNPs was found primarily comparable to the standard reference antifungal drug Hygromycin.

**Table 4:** Antifungal activity of *C. flexuosus* (E.OS1, E.OS2, and E.OS3) essential oil and synthesized MgNPs (MgS1, MgS2, MgS3) against *F. oxysporum*.

Samples	Pathogenic strain (*F. oxysporum*)	
	Fungal growth (mm)	% Age inhibition
Control	79	0
E.OS1	43.66	22.36
E.OS2	42	21.94
E.OS3	44.33	24.05
MgS1	61.33	44.73
MgS2	61.66	46.83
MgS3	60	43.8

### Biosafety of green synthesized magnesium nanoparticles

Biosafety is one of the major concern and MgO nanoparticles have provided it for agriculture, biomedical applications, and most importantly, to the environment by acting as a photocatalyst. MgO is a green synthesis nanoparticle which is generally eco-friendly, less toxic, and cost-effective in comparison with its synthesis from chemicals, which poses major biosafety concerns for the environment. MgO nanoparticles shows antibacterial activity against both gram-positive as well as gram-negative bacteria which make it safe for the environment and mammal cells^39^. In the case of its photocatalytic activity it is generally used to remove a pollutant like Methylene blue from water by acting as a photocatalyst as shown in results.

In terms of toxicity MgO played an important role against breast cancer cell lines (MCF-7)^40^, lung cancer cell lines, and its cytotoxicity test also confirms that these are non-poisonous to healthy RBCs^41^. which shows its biosafety for mammalian cell lines. In the case of agriculture, MgO nanoparticles help in the improvement of biomass construction and plant expansion, which helps in reshaping farming by replacing the use of chemicals like herbicides, pesticides, and fertilizers, which is another biosafety provided through these green synthesized nanoparticles^42^.

## Conclusion

For the first time, the essential oil of *C. flexuosus* plant was utilized as a reducing/capping/stabilizing agent for the fabrication of bio acceptable MgNPs which are endowed with remarkable physicochemical, photocatalytic, antibacterial, and antifungal properties. The well-characterized MgNPs (8–16 nm) are suitable for various advanced application in view of the adopted greener methodology supported by plant-based essential oil using in aqueous medium and devoid of harmful chemicals. Such nanosystems i.e., MgS1 (13.21 nm), MgS2 (12.9 nm), and MgS3 (11.35 nm) exhibited enhanced photocatalytic (91.2%) efficacy against MB dye degradation under the direct sunlight exposure within 3 h. The investigated antibacterial and antifungal performance of MgNPs can efficiently perform against pathogenic bacteria and human fungi. Thus, it is safe to suggest that the involvement of lemongrass oil with a nano system can serve as a natural bio-adsorbent in materials science and such similar strategies can be adopted for other natural, abundant, renewable and safer extractives. The study opens a new window for fabrication of essential oil mediated Mg NPs to develop smart infectious disease combatting strategies owing to their significant antifungal, antibacterial and photocatalytic activities.

## Experimental procedure

### Materials and reagents

Analytical grade reagents and materials, known for production of accurate results, were procured from LobaChemie including magnesium nitrate (MgNO_3_), methylene Blue (MB), and acetone. *C. flexuosous* fresh green leaves were collected from three distinct locations, i.e., site-I (Palampur), site-II (Haryana), site-III (Dehradun). The specimen was deposited in the Herbarium library of Shoolini University, Solan (H.P) under the Voucher No.-SUBMS/BOT-4798. Demineralized water was utilized during all the experiments.

### Synthesis of *C. flexuosus*-based essential oil

The essential oil of *C. flexuosus* was produced by using demineralized water as the extracting medium. The collected leaves were washed twice with distilled water to remove their impurities. In typical extraction, 150 g of fresh leaves of *C. flexuosus* are hydro distilled in 1500 mL of water at 60 °C for 3 h using a Clevenger apparatus; yield of essential oil extracted from site-I, site-II, and site-III being 2.5 mL, 3 mL, and 4 mL, respectively (yielding 1.8%, 2.1%, and 2.5%, respectively). The extracted essential oil is characterized by pale yellow color with a strong lemon-like odor and is stored at 40 °C and was further utilized to fabricate Mg NPs.

### Green synthesis of MgNPs

For biosynthesis of MgNPs, the essential oil is diluted using acetone due to the excellent solubility (2 mL of essential oil in 10 mL of acetone). In a typical synthesis, 7 mL of diluted oil is added to 0.5 M magnesium nitrate, and the solution was kept at 40–60 °C temperature under constant stirring for 2 h. The pH of the solution was maintained by adding 1.5 mL of 0.5 molar NaOH dropwise until pH 7 to attain a pale-yellow suspension. The suspension was kept still for consecutive 5 h. The obtained precipitate was filtered, washed, and dried in an oven at 40 °C for 2 h to obtain Mg NPs which were subjected to various chemical and spectroscopic analyses to confirm their identity. This general processing strategy was adopted for fabricating Mg NP using essential oil from all sites (Palampur S1, Haryana S2, and Dehradun S3).

### Characterization of MgNPs

Various analytical techniques were implemented in the characterization of fabricated biogenic Mg NPs. The synthesis trajectory for the formation of Mg NPs was monitored at different time intervals 1.5 h using a UV–Vis spectrophotometer (Lambda Perkin Elmer), at a wavelength range of 200–700 nm. The functional groups adorning the MG NP's surface and other surface chemical residues were detected using FTIR spectrometer (Perkin Elma) in the spectral range of 650 cm^−1^ to 4000 cm^−1^ with the resolution of 4 cm^−1^. The crystalline phase and crystallite size of fabricated NPs was evaluated using an X-ray diffractometer (X'Pert PRO x-ray diffraction, PANalytical). The observations were recorded deploying Cu-kα radiation (λ = 1.54 Å) in 2θ range of 0–80° using 40kv accelerating voltage. Moreover, the particle size is further confirmed by high-resolution transmission electron microscopy (HR-TEM).

### Photocatalytic activity of MgNPs

The fabricated Mg NPs were used as probe catalysts to investigate the degradation of methylene blue (MB) dye in model synthetic wastewater treatment in the presence of direct sunlight. The experimental methodology includes dispersing 30 mg MgNPs in 2 × 10^−5^ M aqueous solution of dye tainted wastewater at a concentration of 10 ppm of MB. The solution was continuously stirred for 30 min under dark conditions to achieve the absorption–desorption equilibrium of MB dye molecules on the surface of the Mg NPs.

The equilibrated solution was afterward subjected to sunlight for 3 h to observe the photocatalysis of Mg nanoparticles towards MB degradation. After every 15 min interval, 3 mL of the mixture was withdrawn to check the absorbance of dye. The collected samples were centrifuged at 12,000 rpm to separate the nanoparticles, and finally, the resulting supernatant was subjected to UV analysis to evaluate the absorbance.

In the meantime, the dye absorbance efficiency was examined at *λ*max = 665 nm. The photocatalytic efficiency of MgNPs was determined using Eq. (6)^43^.

6 $$\upeta \left( \% \right) = \left[ {\left( {{\text{A}}0{-}{\text{At}}} \right){\text{/A}}0} \right] \times 100$$

where A_0_ and A_t_ were initial and final absorbance after a specific reaction time, respectively.

### Antibacterial assay of MgNPs

The antibacterial activity of fabricated novel MgNPs was assessed against two human pathogens, including *S. aureus* (gram-) [MTCC29213] and *E. coli* (gram +) [MTCC723] using well diffusion assay. They were procured from the Department of Microbiology, Shoolini University, Solan, India. The zone of inhibition was observed as per Kizil et al. 2010 protocol^44^. In Nutrient Broth (NB) medium bacterial strains were conserved. Nutrient Agar was used as the media to culture the bacterial strains. Various concentrations of nanoparticles synthesized from the essential oil of *C. flexuosus* were evaluated against bacterial culture. Strains were swabbed on the surface of the sabouraud agar plates, and wells were prepared from Whatman No. 1 filter paper (diameter of 9 mm). Plates were Lawned with 0.1 OD (Optical Density) of bacterial culture, and wells were made to which 80 μL of the sample was taken from 25 mg/mL stock solution. Ampicillin was used as a positive control for comparing the antibacterial activities and DMSO as a negative control. The plates were incubated at 37 °C for 24 h. The antimicrobial potency of the test samples was measured by determining the diameter of the zones of inhibition in millimeters.

### Antifungal activity of MgNPs

The antifungal activity of fabricated novel MgNPs was assessed against human pathogen, *F. oxysporum* (MTCC9913), using a well diffusion assay. They were procured from the Department of Microbiology, Shoolini University, Solan, India. The zone of inhibition was observed as per Gavilanes-Martínez et al. 2021 protocol^45^. In Potato Dextrose Broth (PDB) media, fungal strains were conserved. Potato Dextrose Agar (PDA) media was used as the media for the culturing of fungal strains. In a typical process, initially, PDB media was taken into glass test tubes. After autoclaving, the fungus bits were kept in the glass tubes in Laminar Air Flow. Plates were cultured, and wells were made to which 60 μL of the sample was taken from 5 mg/mL stock solution. For comparing the antifungal activities, Hygromycin was taken as the positive control. The plates were incubated at 27 °C for 6 days. The antifungal potency of the test samples was measured by determining the diameter of the zones of inhibition in millimeters.

## Data Availability

All data generated and analyzed during this study are included in this paper.
